# Exploratory examination of the association between physical-mental multimorbidity and physical activity in children

**DOI:** 10.3389/fped.2023.920629

**Published:** 2023-02-02

**Authors:** Chloe Bedard, Brian W. Timmons, Mark A. Ferro

**Affiliations:** ^1^ School of Public Health Sciences, University of Waterloo, Waterloo, ON, Canada; ^2^ Child Health & Exercise Medicine Program, McMaster University, Hamilton, ON, Canada

**Keywords:** physical activity, multimorbidity (MM), children, youth, mental illness

## Abstract

Children with physical illnesses often experience co-occurring mental illness (known as multimorbidity; MM) and it is currently unknown if MM is associated with physical activity (PA) and if the association differs between internalizing and externalizing disorders. Therefore, the aim of this study was to examine the association between MM and PA. Baseline data from the Multimorbidity in Children and Youth Across the Life Course (MY LIFE) cohort was used. MY LIFE is an ongoing prospective study that follows children ages 2 to 16 years with a chronic physical illness and measures PA using accelerometry and mental illness using the Mini International Neuropsychiatric Interview for Children and Adolescents. 140 children (53.2%) provided valid accelerometer data. Children with internalizing disorders recorded less light (B = −5.87), moderate (B = −1.82), and vigorous PA (B = −1.93) and fewer days meeting PA guidelines [Exp(B) = 0.73] and those with externalizing disorders recorded more light (B = 4.85), moderate (B = 1.78), and vigorous PA (B = 2.41) and more days meeting PA guidelines [Exp(B) = 1.06]. However, only the association between internalizing disorder and days meeting PA guidelines was statistically significant. This study provides preliminary evidence that children with MM may accumulate less PA depending on the type of mental illness they experience.

## 1. Introduction

Physical activity (PA) results in several positive physical and mental health benefits for both adults and children (1, 2). Evidence in children shows that PA contributes to healthy body weight, cardiovascular and muscular fitness, bone mineral density, healthy cholesterol, blood lipids, and blood pressure, as well as decreases symptoms of depression and anxiety, and can improve self-esteem (2–4). Multiple national and international organizations have evidence-informed daily PA guidelines that recommend children ages 5-to-17 years of age engage in at least 60 min of moderate -to-vigorous PA (MVPA) and children 2 to 4 years should be active at any intensity for at least 180 min (5, 6). Unfortunately, 67%–80% of children worldwide do not meet these recommendations (7, 8). This is particularly concerning given that health habits formed in childhood track consistently into adulthood, leading to prolonged exposure to the negative consequences of physical inactivity (9). Furthermore, emerging evidence suggests that PA engagement during adolescence has a lasting effect on the risk of developing multiple health concerns in middle adulthood (10). Therefore, it is critical that the patterns and correlates of PA early in life are examined to determine effective methods to promote PA to enable children to reap these positive physical and mental health benefits.

Surveillance of PA has typically excluded children and adolescents with chronic physical illness, leading to evidence on PA levels for these children to be less robust (11). However, prevalence estimates suggest that 1 in 4 children has a chronic physical illness (12), warranting specific investigation into their risk of physical inactivity. Recent estimates indicate that children with chronic physical illnesses are similarly inactive compared with healthy controls (11), but there is a gap in the literature regarding possible reasons for physical inactivity among these children. Some evidence suggests that functional limitations due to their physical illness may prohibit engagement in PA (13). Additionally, upwards of 50% of children with a physical illness experience co-occurring mental illness (known as multimorbidity, MM) (14). It is established that children with mental illnesses often experience low levels of PA (15), however, the deficit may depend on the type of mental illness. For example, children with an externalizing disorder such as attention-deficit hyperactivity disorder have been reported to show increased unorganized PA (16), whereas adolescents with internalizing disorders (e.g., depressive symptoms) have been shown to have lower levels of participation in PA (17). It is unknown if MM is associated with PA in children.

The objective of this study was to examine the association between MM and PA in children and determine whether the association differs in those with an internalizing or externalizing mental illness; it is hypothesized that children with internalizing disorders will engage in less PA and those with externalizing behaviours will engage in more PA.

## 2. Methods

### 2.1. Participants

Baseline data from the Multimorbidity in Children and Youth across the Life Course (MY LIFE) study was used for this analysis. This prospective cohort study follows 263 children ages 2 to 16 years (mean age = 9.42, SD = 3.98) with a physician-diagnosed chronic physical illness (defined as a condition that has or is expected to last ≥12 months with functional limitations, dependencies, and the need for additional healthcare; including dermatological, endocrine, gastroenterological, hematological, neurological, respiratory, and rheumatological disorders). Participants were recruited from outpatient clinics at McMaster Children's Hospital and were required to be able to and have parents able to communicate in English. The sampling procedures and description of the full cohort have been previously published (18, 19).

### 2.2. Study participants

Eligible and consenting families were scheduled to complete baseline appointments that were approximately 60–90 min in length with subsequent follow up at 6, 12, and 24 months. Baseline assessments were conducted from August 2017 to November 2019. Computer-assisted self-report questionnaires were administered to all parents and participants over the age of 10 years. Trained research assistants also collected biological samples, anthropometric data, and fitted each participant with an accelerometer. Written consent was obtained from all parents and participants over 16 years; assent was collected from participants ages 7–15 years. Ethical approval for this study was obtained from the Hamilton Integrated Research Ethical Board and the University of Waterloo Human Research Ethics Board.

### 2.3. Measures

#### 2.3.1. Mental illness

To determine the presence of multimorbidity (MM), all parents (over the telephone) and participants over 10 years of age (in-person) were asked to complete the Mini International Neuropsychiatric Interview for Children and Adolescents (MINI-KID) (20). The MINI-KID contains modules aligned with the diagnostic criteria of the DSM-5 and ICD-10 to detect internalizing disorders (i.e., major depressive episode, separation anxiety disorder, social anxiety disorder, specific phobias, and generalized anxiety disorder) and externalizing disorders (i.e., attention-deficit hyperactivity disorder, conduct disorder, and oppositional defiant disorder). The MINI-KID has excellent psychometric properties among children from clinical settings (21–24). Participants were categorized as MM if they screened positive for at least one mental illness; if they did not screen positive for any mental illness, they were categorized as not MM (physical illness only).

#### 2.3.2. Physical activity

Participants were asked to wear an Actigraph GT3X accelerometer fitted around their waist to be worn over their right hip for 7 consecutive days (25). Participants and their parents were instructed to remove the device only during sleep and water-based activities and a logbook was provided to each family to record wear-time. Three second epochs were used to record physical activity (26). The Actilife software (Actigraph) was used to clean and process the accelerometer data. Data were considered valid if at least 10 h of wear-time were recorded on 3 days; non-wear time was defined as >60 min of consecutive zero counts or when the logbook indicated the device was removed (27, 28). The Evenson cut points were used to define PA intensity of light, moderate, and vigorous as >100, ≥2,296, ≥4,012 counts per minute, respectively (29). The number of days children met PA guidelines for their respective age categories was also computed. Guidelines for children <5 years state that average daily total PA should be at least 180 min with 3 to 4 year olds requiring at least an average daily of 60 min of MVPA (30). Guidelines for children 5 to 17 years state that they should engage in average daily 60 min of MVPA (30). Given the common guidance related to MVPA, we focused on this PA characteristic in our analyses.

#### 2.3.3. Covariates

Parents were asked to complete the 12-item World Health Organization Disability Assessment Schedule (WHODAS) 2.0 to determine the level of physical functioning of their child (31). Self-report questionnaire for parents included items from population-based surveys conducted by Statistics Canada on various demographic information including household income before taxes, and age and sex, relating to both themselves and their child. Body mass index (BMI) was computed using participants' standing height and weight and percentiles were calculated with the World Health Organization growth reference data for children (32).

### 2.4. Statistical analyses

Using data only from participants with valid accelerometer data, multiple regression models were built with average daily minutes spent in each intensity of PA (light, moderate, and vigorous PA) as dependent variables. Average daily minutes of PA in each intensity were computed as the sum of the minutes of PA recorded on each valid day of wear divided by the number of valid days of wear. Three regression models were built separately using MM status (any mental illness vs. no mental illness), presence of internalizing disorder (vs. no internalizing disorder), and presence of externalizing disorder (vs. no externalizing disorder), respectively, as the independent variables. Three Poisson regression models with the number of days meeting PA guidelines as the dependent variable were also conducted. All models were adjusted for the number of valid days of accelerometer wear, age (<10 years and ≥10 years) sex, household income, WHODAS 2.0 scores, and BMI percentile. All statistical analyses were performed in SPSS (33) with *α* < .05. SPSS model syntax and assumption testing results are provided in a Supplementary file.

### 2.5. Missing data

Valid accelerometer data were obtained from 140 of the 263 total children (53.2%) enrolled in the study at baseline. Among those with valid accelerometer data there were nine children who did not have height and/or weight data available to compute BMI, and therefore were removed from the analyses. Chi-square tests showed that the proportion of missing accelerometer and BMI data was not different between males and females, between those <10 years or ≥10 years of age, or by MM status.

## 3. Results

Children in the analytical sample (i.e., valid accelerometer data) were comparable to the total sample with respect to demographic and clinical characteristics (see Table 1). On average, children spent majority of their PA in light PA, with decreasing amounts of time spent in higher intensities of PA (see Table 1). Table 2 shows that of children without MM, just under half had a daily average MVPA of >1 h, averaging 73.34 min. Of children with MM, only 38% engaged in >1 h of MVPA daily, averaging 79.77 min.

**Table 1:** Descriptive statistics of the baseline sample with valid accelerometer data.

Variable	Total (*n* = 263)	With Valid Accelerometer Data (*n* = 140)
Child's Age in years; *mean* (SD)	9.42 (4.20)	9.30 (3.76)
Female; *n* (%)	124 (47.1%)	72 (51.4%)
Household Income <$119,000; *n* (%)	139 (52.9%)	73 (52.1%)
Dermatological; *n* (%)	23 (8.7)	8 (5.7)
Endocrine; *n* (%)	38 (14.4)	20 (14.3)
Gastrological; *n* (%)	34 (12.9)	18 (12.9)
Hematological; *n* (%)	29 (11)	15 (10.7)
Neurological; *n* (%)	12 (4.6)	7 (5.0)
Respiratory; *n* (%)	54 (20.5)	29 (20.7)
Rheumatological; *n* (%)	73 (27.8)	43 (30.7)
Average Daily LPA (min); *mean* (SD)	–	165.16 (43.26)
Average Daily MPA (min); *mean* (SD)	–	32.45 (9.86)
Average Daily VPA (min); *mean* (SD)	–	23.76 (12.48)
Average Daily total PA (min); *mean* (SD)	–	221.37 (59.60)
Any Internalizing Disorder; *n* (%)	76 (28.9)	38 (27.1)
Any Externalizing Disorder; *n* (%)	42 (16.0)	26 (18.6)
MM (any mental illness); *n* (%)	100 (38.0)	55 (39.3)

*SD* = standard deviations; *n* = sample size.

**Table 2:** Description of daily MVPA patterns stratified by MM Status.

	No MM	MM
< 30 min Average daily MVPA		
*n* (%)	10 (11.8)	5 (9.1)
Mean MVPA (SD)	22.69 (4.53)	20.25 (4.49)
30–60 min Average daily MVPA		
*n* (%)	35 (41.2)	29 (52.7)
Mean MVPA (SD)	45.64 (7.11)	46.07 (8.27)
>60 min Average daily MVPA		
*n* (%)	40 (47.1)	21 (38.2)
Mean MVPA (SD)	73.34 (11.33)	79.77 (14.88)

*MM,* multimorbidity; *MVPA,* Moderate-to-vigorous physical activity; *SD,* standard deviation; *n*: number of participants; *%:* percentage.

The regression analyses (Table 3) show that children with MM had non-significantly fewer number of days meeting PA guidelines, and non-significantly more minutes of LPA, MPA and VPA. Children with internalizing disorders had non-significantly fewer minutes of all intensities of PA, as well as fewer days meeting PA guidelines, whereas those with externalizing disorders had non-significantly more minutes of all intensities of PA and non-significantly more days meeting PA guidelines. Only the association between internalizing disorder and days meeting PA guidelines was statistically significant.

**Table 3:** Regression models of MM Status and PA.

	LPA	MPA	VPA	Days Meeting Guidelines
Variable	Beta (95% CI)	Beta (95% CI)	Beta (95% CI)	Exp(B) (95% CI)
MM (any mental illness)	0.22 (−13.78 to 14.22)	0.93 (−2.76 to 4.61)	1.56 (−3.13 to 6.26)	0.87 (0.68 to 1.12)
Any Internalizing	−5.87 (−20.87 to 9.13)	−1.82 (−5.77 to 2.13)	−1.93 (−6.98 to 3.11)	0.73 (0.54 to 0.98)
Any Externalizing	4.85 (−11.91 to 21.59)	1.78 (−2.63 to 6.19)	2.41 (−3.22 to 8.03)	1.06 (0.78 to 1.40)

*LPA,* light physical activity; *MPA,* moderate physical activity; *VPA,* vigorous physical activity; Adjusted for number of valid days of wear, age (≥ 10 years), sex (female), household income, WHODAS, and body mass index percentile.

## 4. Discussion

The study examined the association between the presence of MM and PA, and whether this association differed as a function of the type of mental illness, in children. While associations were not statistically significant, the pattern of results suggest that MM may be associated with fewer days meeting the recommended levels of PA. Among those engaging in >60 min of daily MVPA, there are fewer children with MM than those without; however, the former has a higher average MVPA compared to those without MM. These results suggest that children with MM may be less likely to reach daily recommended levels of MVPA, however on days they do, their activity levels are high. Moreover, when categorizing MM as the presence of either an internalizing or externalizing behaviour, the results suggest a divergent association with PA, with internalizing disorders non-significantly associated with less PA and externalizing disorders non-significantly associated with more PA. Therefore, the null positive association between any MM and minutes engaged in PA is likely obscured due to the opposite associations with internalizing and externalizing disorders.

Children with chronic physical illnesses have been reported as similarly inactive compared to their healthy peers (11), however to our knowledge there are no studies comparing the PA levels of children with physical illness to those with multimorbidity. The results of this study suggest that children with MM have similar average levels of PA, however, may accumulate these minutes in different intensities and across fewer days in the week and this may depend on the type of mental illness they experience. Reasons for children with MM meeting recommended MVPA on fewer days of the week are unclear. The complexities of their health may result in days in which children have low energy and motivation and even light PA may feel like a challenge, whereas other days a positive mood allows them to expend high amounts of energy. This may explain why children with internalizing disorder were less likely to accumulate sufficient daily PA. Evidence shows that adolescents with mood or anxiety disorders may feel tired and/or disinterested in participating in PA and may also prefer to avoid the social aspects of PA (3). Children experiencing internalizing disorders may have fewer days on which the psychopathology of their disorders does not impact their motivation and energy to engage in PA. Whereas externalizing disorders are typically characterized by hyperkinetic behaviours (e.g., running or climbing in inappropriate situations), and therefore daily PA may be higher. However, it is important to note that PA engaged in through hyperkinetic behaviours may be unorganized as it has been shown that children with externalizing disorders do not report high levels of participation in sport or intentional PA (15). Alternatively, socio-environmental factors may influence PA levels such as overprotective care environments, lack of access/opportunities for PA, or knowledge- and self-efficacy-barriers regarding safe and appropriate types of PA. It is worth noting that in a sample of 13–17 year old children, Mangerud (15) found that youth with mental illnesses (both internalizing and externalizing) were less active compared to their healthy peers. Therefore, these socio-environmental factors may operate similarly on both children with a physical illness and those with MM, such that it reduces the differential risk of inactivity. Furthermore, there are likely additional barriers known to constrain PA of children without illness that may be similarly relevant to children with MM such as motor competence, self-image, and peer and parental support for PA (15, 34, 35).

We believe that there are currently no other studies examining the association between PA and MM among children. Strengths of this study include the objective measurement of PA using accelerometery, negating the recall and social desirability biases inherent in self-report (36). MY LIFE also implemented the MINI-KID assessment to provide a reliable and valid assessment of mental illness aligned with clinical diagnoses, thereby providing a more robust determination of MM beyond symptomology. Additionally, participants were sampled across a range physical illness to increase the generalizability of the sample.

However, methodological weaknesses must also be acknowledged. Most limiting is the analytic sample size, precluding conclusions based on the model results; therefore, the analyses are exploratory and hypothesis-generating. Also, the data do not provide insight into the context or type of PA. It will be important to implement measures of qualitative assessments of PA concurrently with accelerometery to understand the types of PA children are engaging in. Finally, this study did not include a comparison of children without a physical illness, therefore it remains unclear how the presence of a mental illness is associated with PA when the child does not have a physical illness.

### 4.1. Conclusions and future directions

Children with and without MM have similar average levels of PA; however, may accumulate these minutes in different intensities and across fewer days in the week and this depends on the type of mental illness they experience. These results are strictly exploratory, however they provide support to further study the complex effect of MM on PA engagement to determine if these associations are robust.

## Data Availability

The datasets presented in this article are not readily available because research ethical approval for this study does not permit the public sharing of study data. However, requests to access data should be made in writing by contacting the corresponding author by email to initiate discussions regarding potential research projects.
