# Supplementary material for: Exploratory examination of the association between physical-mental multimorbidity and physical activity in children
Source: Front Pediatr. 2023 Feb 2;11:920629. doi: 10.3389/fped.2023.920629 (PMC9932503; doi:10.3389/fped.2023.920629)
Supplement: Supplementary file 1 [file Table1.docx]

Supplementary Information: SPSS Code and Assumption Testing

REGRESSION

/MISSING LISTWISE

/STATISTICS COEFF OUTS CI(95) R ANOVA COLLIN TOL

/CRITERIA=PIN(.05) POUT(.10)

/NOORIGIN

**/DEPENDENT Evenson_Light**

**/METHOD=ENTER pwhodastotal1 BMI_percentile pincome1 gender Valid_Days Binary_age_10 panydx1**

/SCATTERPLOT=(*SDRESID ,*ADJPRED)

/RESIDUALS HISTOGRAM(ZRESID) NORMPROB(ZRESID).

| **Model Summary^b^** | | | | |
| --- | --- | --- | --- | --- |
| Model | R | R Square | Adjusted R Square | Std. Error of the Estimate |
| 1 | .667^a^ | .445 | .414 | 33.833341022567550 |
| a. Predictors: (Constant), Parent MINI: Screen Positive to ANY disorder?, Number of Valid Days of Wear, BMI_percentile, 10 years and older, Parent's income category (before taxes), child gender, Parent total WHODAS score | | | | |
| b. Dependent Variable: Evenson Cutpoint for Light | | | | |

| **ANOVA^a^** | | | | | | |
| --- | --- | --- | --- | --- | --- | --- |
| Model | | Sum of Squares | df | Mean Square | F | Sig. |
| 1 | Regression | 113023.397 | 7 | 16146.200 | 14.105 | .000^b^ |
|  | Residual | 140797.481 | 123 | 1144.695 |  |  |
|  | Total | 253820.878 | 130 |  |  |  |
| a. Dependent Variable: Evenson Cutpoint for Light | | | | | | |
| b. Predictors: (Constant), Parent MINI: Screen Positive to ANY disorder?, Number of Valid Days of Wear, BMI_percentile, 10 years and older, Parent's income category (before taxes), child gender, Parent total WHODAS score | | | | | | |

| Model | | Collinearity Statistics | |
| --- | --- | --- | --- |
|  |  | Tolerance | VIF |
| 1 | (Constant) |  |  |
|  | Parent total WHODAS score | .609 | 1.641 |
|  | BMI_percentile | .831 | 1.204 |
|  | Parent's income category (before taxes) | .791 | 1.264 |
|  | child gender | .883 | 1.132 |
|  | Number of Valid Days of Wear | .922 | 1.084 |
|  | 10 years and older | .957 | 1.045 |
|  | Parent MINI: Screen Positive to ANY disorder? | .741 | 1.350 |

**Charts**


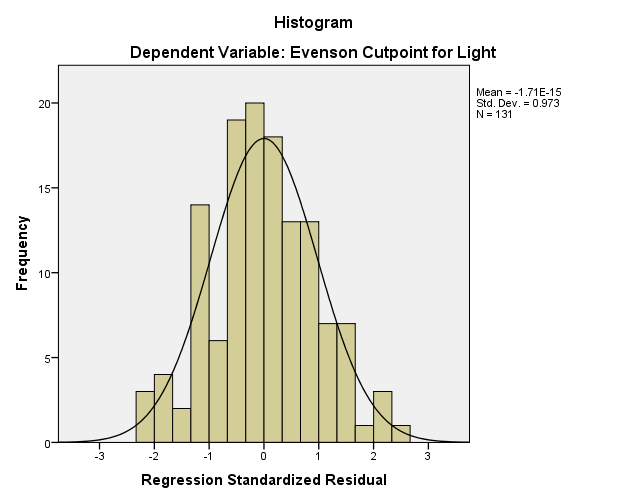


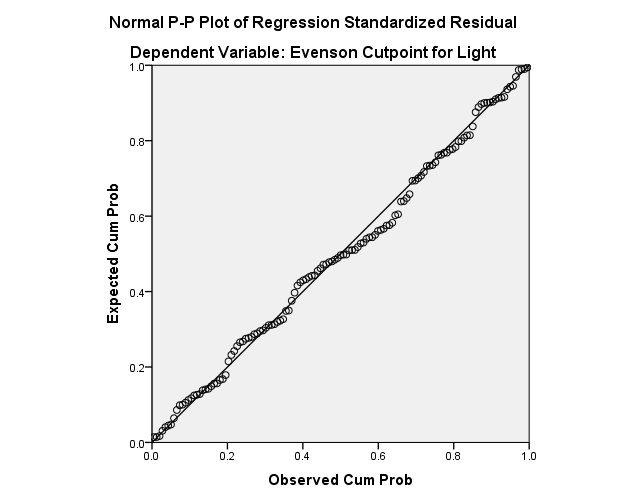


REGRESSION

/MISSING LISTWISE

/STATISTICS COEFF OUTS CI(95) R ANOVA COLLIN TOL

/CRITERIA=PIN(.05) POUT(.10)

/NOORIGIN

**/DEPENDENT Evenson_Moderate**

**/METHOD=ENTER pwhodastotal1 BMI_percentile pincome1 gender Valid_Days Binary_age_10 panydx1**

/SCATTERPLOT=(*SDRESID ,*ADJPRED)

/RESIDUALS HISTOGRAM(ZRESID) NORMPROB(ZRESID).

| **Model Summary^b^** | | | | |
| --- | --- | --- | --- | --- |
| Model | R | R Square | Adjusted R Square | Std. Error of the Estimate |
| 1 | .489^a^ | .239 | .196 | 8.907342281385002 |
| a. Predictors: (Constant), Parent MINI: Screen Positive to ANY disorder?, Number of Valid Days of Wear, BMI_percentile, 10 years and older, Parent's income category (before taxes), child gender, Parent total WHODAS score | | | | |
| b. Dependent Variable: Evenson Cutpoint for Moderate | | | | |

| **ANOVA^a^** | | | | | | |
| --- | --- | --- | --- | --- | --- | --- |
| Model | | Sum of Squares | df | Mean Square | F | Sig. |
| 1 | Regression | 3066.893 | 7 | 438.128 | 5.522 | .000^b^ |
|  | Residual | 9758.912 | 123 | 79.341 |  |  |
|  | Total | 12825.804 | 130 |  |  |  |
| a. Dependent Variable: Evenson Cutpoint for Moderate | | | | | | |
| b. Predictors: (Constant), Parent MINI: Screen Positive to ANY disorder?, Number of Valid Days of Wear, BMI_percentile, 10 years and older, Parent's income category (before taxes), child gender, Parent total WHODAS score | | | | | | |

| Model | | Collinearity Statistics | |
| --- | --- | --- | --- |
|  |  | Tolerance | VIF |
| 1 | (Constant) |  |  |
|  | Parent total WHODAS score | .609 | 1.641 |
|  | BMI_percentile | .831 | 1.204 |
|  | Parent's income category (before taxes) | .791 | 1.264 |
|  | child gender | .883 | 1.132 |
|  | Number of Valid Days of Wear | .922 | 1.084 |
|  | 10 years and older | .957 | 1.045 |
|  | Parent MINI: Screen Positive to ANY disorder? | .741 | 1.350 |

**Charts**


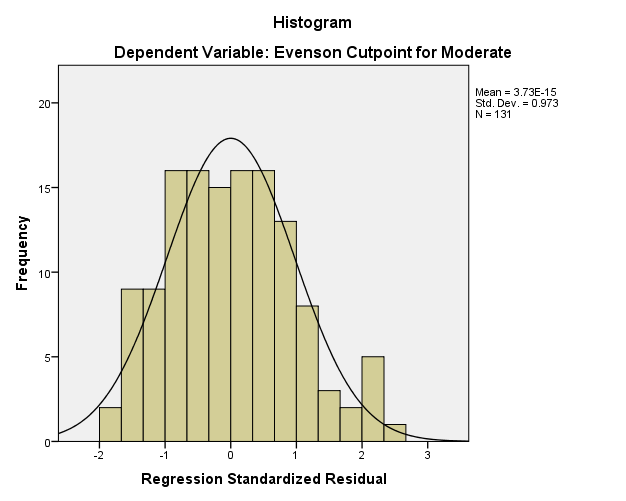


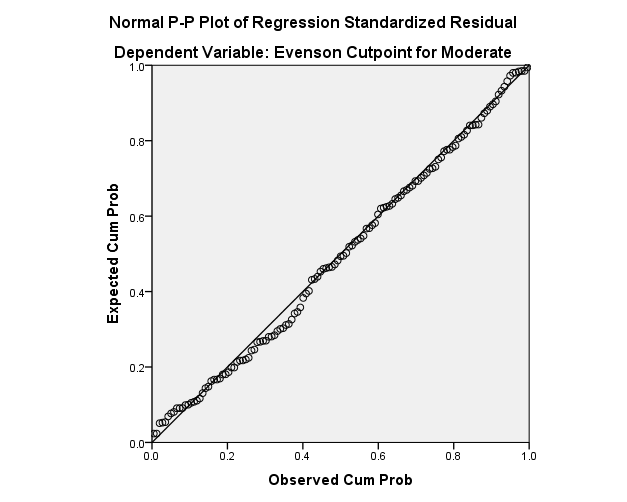


REGRESSION

/MISSING LISTWISE

/STATISTICS COEFF OUTS CI(95) R ANOVA COLLIN TOL

/CRITERIA=PIN(.05) POUT(.10)

/NOORIGIN

**/DEPENDENT Evenson_Vigorous**

**/METHOD=ENTER pwhodastotal1 BMI_percentile pincome1 gender Valid_Days Binary_age_10 panydx1**

/SCATTERPLOT=(*SDRESID ,*ADJPRED)

/RESIDUALS HISTOGRAM(ZRESID) NORMPROB(ZRESID).

| **Model Summary^b^** | | | | |
| --- | --- | --- | --- | --- |
| Model | R | R Square | Adjusted R Square | Std. Error of the Estimate |
| 1 | .484^a^ | .234 | .191 | 11.358068841957424 |
| a. Predictors: (Constant), Parent MINI: Screen Positive to ANY disorder?, Number of Valid Days of Wear, BMI_percentile, 10 years and older, Parent's income category (before taxes), child gender, Parent total WHODAS score | | | | |
| b. Dependent Variable: Evenson Cutpoint for Vigorous | | | | |

| **ANOVA^a^** | | | | | | |
| --- | --- | --- | --- | --- | --- | --- |
| Model | | Sum of Squares | df | Mean Square | F | Sig. |
| 1 | Regression | 4859.286 | 7 | 694.184 | 5.381 | .000^b^ |
|  | Residual | 15867.705 | 123 | 129.006 |  |  |
|  | Total | 20726.991 | 130 |  |  |  |
| a. Dependent Variable: Evenson Cutpoint for Vigorous | | | | | | |
| b. Predictors: (Constant), Parent MINI: Screen Positive to ANY disorder?, Number of Valid Days of Wear, BMI_percentile, 10 years and older, Parent's income category (before taxes), child gender, Parent total WHODAS score | | | | | | |

| Model | | Collinearity Statistics | |
| --- | --- | --- | --- |
|  |  | Tolerance | VIF |
| 1 | (Constant) |  |  |
|  | Parent total WHODAS score | .609 | 1.641 |
|  | BMI_percentile | .831 | 1.204 |
|  | Parent's income category (before taxes) | .791 | 1.264 |
|  | child gender | .883 | 1.132 |
|  | Number of Valid Days of Wear | .922 | 1.084 |
|  | 10 years and older | .957 | 1.045 |
|  | Parent MINI: Screen Positive to ANY disorder? | .741 | 1.350 |

**Charts**


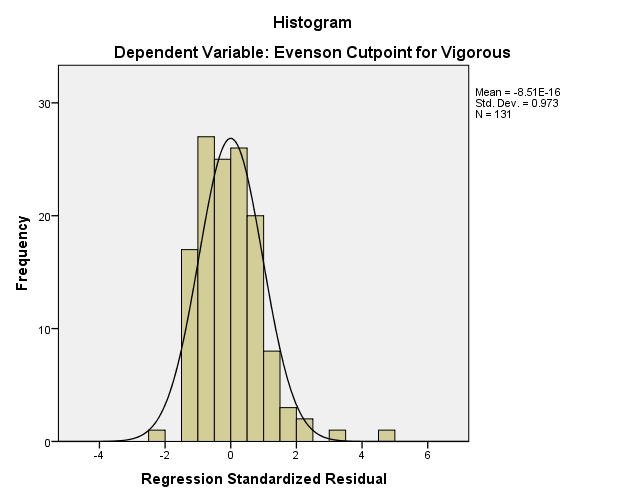


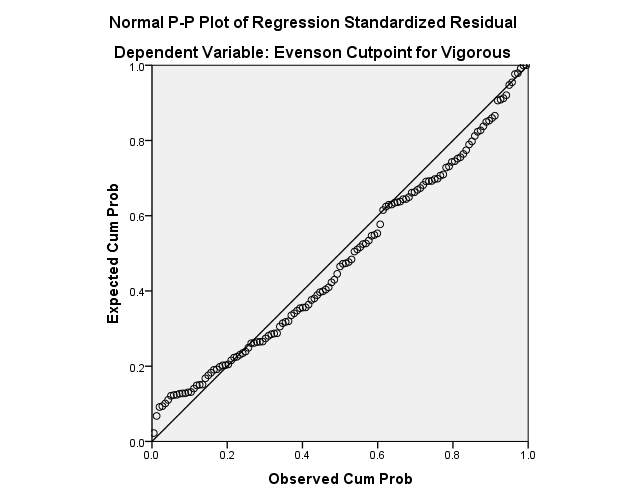


* Generalized Linear Models.

**GENLIN Num_days_Guidelines BY panydx1 gender Binary_age_10 (ORDER=DESCENDING) WITH Valid_Days pwhodastotal1 pincome1 BMI_percentile**

/MODEL gender Binary_age_10 Valid_Days pwhodastotal1 pincome1 BMI_percentile panydx1 INTERCEPT=YES

DISTRIBUTION=POISSON LINK=LOG

/CRITERIA METHOD=FISHER SCALE=1 COVB=MODEL MAXITERATIONS=100 MAXSTEPHALVING=5 PCONVERGE=1E-006(ABSOLUTE) SINGULAR=1E-012 ANALYSISTYPE=3(WALD) CILEVEL=95 CITYPE=WALD LIKELIHOOD=FULL

/MISSING CLASSMISSING=EXCLUDE

/PRINT CPS DESCRIPTIVES MODELINFO FIT SUMMARY SOLUTION (EXPONENTIATED).

| **Model Information** | |
| --- | --- |
| Dependent Variable | Num_days_Guidelines |
| Probability Distribution | Poisson |
| Link Function | Log |

| **Omnibus Test^a^** | | |
| --- | --- | --- |
| Likelihood Ratio Chi-Square | df | Sig. |
| 106.190 | 7 | .000 |
| Dependent Variable: Num_days_Guidelines  Model: (Intercept), gender, Binary_age_10, Valid_Days, pwhodastotal1, pincome1, BMI_percentile, panydx1 | | |
| a. Compares the fitted model against the intercept-only model. | | |

REGRESSION

/MISSING LISTWISE

/STATISTICS COEFF OUTS CI(95) R ANOVA

/CRITERIA=PIN(.05) POUT(.10)

/NOORIGIN

**/DEPENDENT Evenson_Light**

**/METHOD=ENTER Internalizing Binary_age_10 Valid_Days gender pincome1 pwhodastotal1 BMI_percentile**

/SCATTERPLOT=(*SDRESID ,*ADJPRED)

/RESIDUALS HISTOGRAM(ZRESID) NORMPROB(ZRESID).

| **Model Summary** | | | | |
| --- | --- | --- | --- | --- |
| Model | R | R Square | Adjusted R Square | Std. Error of the Estimate |
| 1 | .669^a^ | .448 | .417 | 33.751172282854170 |
| a. Predictors: (Constant), BMI_percentile, Number of Valid Days of Wear, Internalizing, 10 years and older, child gender, Parent's income category (before taxes), Parent total WHODAS score | | | | |

| **ANOVA^a^** | | | | | | |
| --- | --- | --- | --- | --- | --- | --- |
| Model | | Sum of Squares | df | Mean Square | F | Sig. |
| 1 | Regression | 113706.457 | 7 | 16243.780 | 14.260 | .000^b^ |
|  | Residual | 140114.421 | 123 | 1139.142 |  |  |
|  | Total | 253820.878 | 130 |  |  |  |
| a. Dependent Variable: Evenson Cutpoint for Light | | | | | | |
| b. Predictors: (Constant), BMI_percentile, Number of Valid Days of Wear, Internalizing, 10 years and older, child gender, Parent's income category (before taxes), Parent total WHODAS score | | | | | | |

| Model | | Collinearity Statistics | | |
| --- | --- | --- | --- | --- |
|  |  | Tolerance | VIF |  |
| 1 | (Constant) |  |  |  |
|  | Parent total WHODAS score | .625 | 1.600 |  |
|  | BMI_percentile | .830 | 1.205 |  |
|  | Parent's income category (before taxes) | .790 | 1.265 |  |
|  | child gender | .923 | 1.083 |  |
|  | Number of Valid Days of Wear | .925 | 1.081 |  |
|  | 10 years and older | .949 | 1.053 |  |
|  | Internalizing | .788 | 1.268 |  |


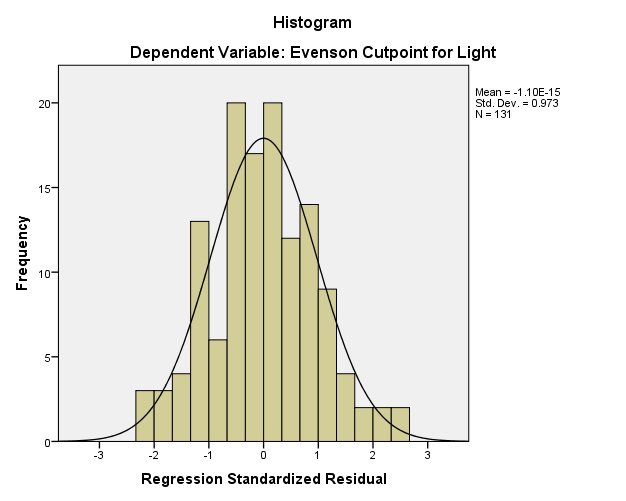


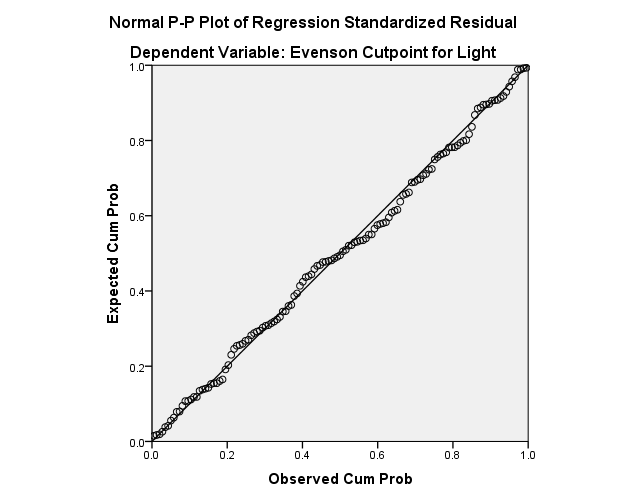


REGRESSION

/MISSING LISTWISE

/STATISTICS COEFF OUTS CI(95) R ANOVA

/CRITERIA=PIN(.05) POUT(.10)

/NOORIGIN

**/DEPENDENT Evenson_Moderate**

**/METHOD=ENTER Internalizing Binary_age_10 Valid_Days gender pincome1 pwhodastotal1 BMI_percentile**

/SCATTERPLOT=(*SDRESID ,*ADJPRED)

/RESIDUALS HISTOGRAM(ZRESID) NORMPROB(ZRESID).

| **Model Summary** | | | | |
| --- | --- | --- | --- | --- |
| Model | R | R Square | Adjusted R Square | Std. Error of the Estimate |
| 1 | .493^a^ | .243 | .200 | 8.886171908904513 |
| a. Predictors: (Constant), BMI_percentile, Number of Valid Days of Wear, Internalizing, 10 years and older, child gender, Parent's income category (before taxes), Parent total WHODAS score | | | | |

| **ANOVA^a^** | | | | | | |
| --- | --- | --- | --- | --- | --- | --- |
| Model | | Sum of Squares | df | Mean Square | F | Sig. |
| 1 | Regression | 3113.226 | 7 | 444.747 | 5.632 | .000^b^ |
|  | Residual | 9712.578 | 123 | 78.964 |  |  |
|  | Total | 12825.804 | 130 |  |  |  |
| a. Dependent Variable: Evenson Cutpoint for Moderate | | | | | | |
| b. Predictors: (Constant), BMI_percentile, Number of Valid Days of Wear, Internalizing, 10 years and older, child gender, Parent's income category (before taxes), Parent total WHODAS score | | | | | | |

| Model | | Collinearity Statistics | |
| --- | --- | --- | --- |
|  |  | Tolerance | VIF |
| 1 | (Constant) |  |  |
|  | Parent total WHODAS score | .625 | 1.600 |
|  | BMI_percentile | .830 | 1.205 |
|  | Parent's income category (before taxes) | .790 | 1.265 |
|  | child gender | .923 | 1.083 |
|  | Number of Valid Days of Wear | .925 | 1.081 |
|  | 10 years and older | .949 | 1.053 |
|  | Internalizing | .788 | 1.268 |


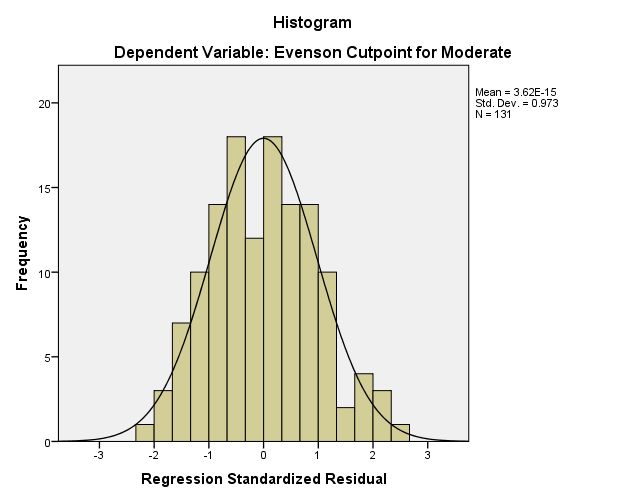


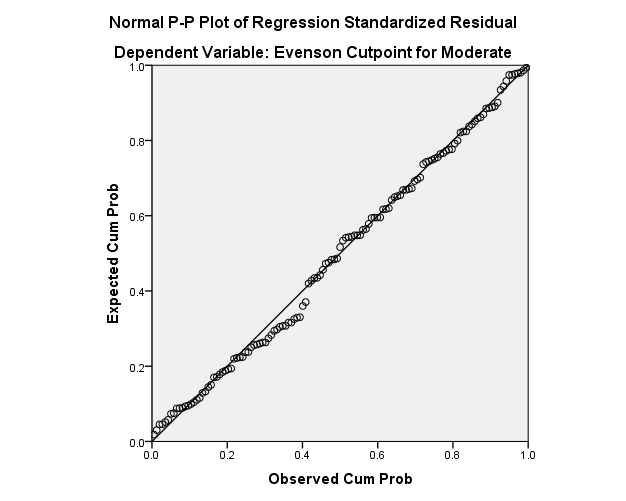


REGRESSION

/MISSING LISTWISE

/STATISTICS COEFF OUTS CI(95) R ANOVA

/CRITERIA=PIN(.05) POUT(.10)

/NOORIGIN

**/DEPENDENT Evenson_Vigorous**

**/METHOD=ENTER Internalizing Binary_age_10 Valid_Days gender pincome1 pwhodastotal1 BMI_percentile**

/SCATTERPLOT=(*SDRESID ,*ADJPRED)

/RESIDUALS HISTOGRAM(ZRESID) NORMPROB(ZRESID).

| **Model Summary** | | | | |
| --- | --- | --- | --- | --- |
| Model | R | R Square | Adjusted R Square | Std. Error of the Estimate |
| 1 | .485^a^ | .235 | .192 | 11.351562204683978 |
| a. Predictors: (Constant), BMI_percentile, Number of Valid Days of Wear, Internalizing, 10 years and older, child gender, Parent's income category (before taxes), Parent total WHODAS score | | | | |

| **ANOVA^a^** | | | | | | |
| --- | --- | --- | --- | --- | --- | --- |
| Model | | Sum of Squares | df | Mean Square | F | Sig. |
| 1 | Regression | 4877.461 | 7 | 696.780 | 5.407 | .000^b^ |
|  | Residual | 15849.530 | 123 | 128.858 |  |  |
|  | Total | 20726.991 | 130 |  |  |  |
| a. Dependent Variable: Evenson Cutpoint for Vigorous | | | | | | |
| b. Predictors: (Constant), BMI_percentile, Number of Valid Days of Wear, Internalizing, 10 years and older, child gender, Parent's income category (before taxes), Parent total WHODAS score | | | | | | |

| Model | | Collinearity Statistics | |
| --- | --- | --- | --- |
|  |  | Tolerance | VIF |
| 1 | (Constant) |  |  |
|  | Parent total WHODAS score | .625 | 1.600 |
|  | BMI_percentile | .830 | 1.205 |
|  | Parent's income category (before taxes) | .790 | 1.265 |
|  | child gender | .923 | 1.083 |
|  | Number of Valid Days of Wear | .925 | 1.081 |
|  | 10 years and older | .949 | 1.053 |
|  | Internalizing | .788 | 1.268 |


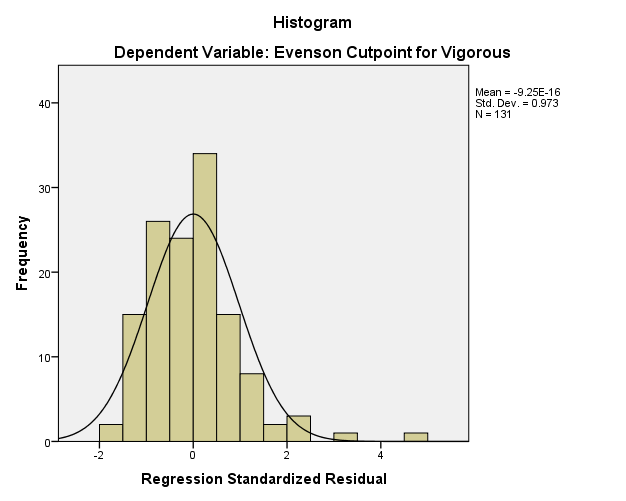


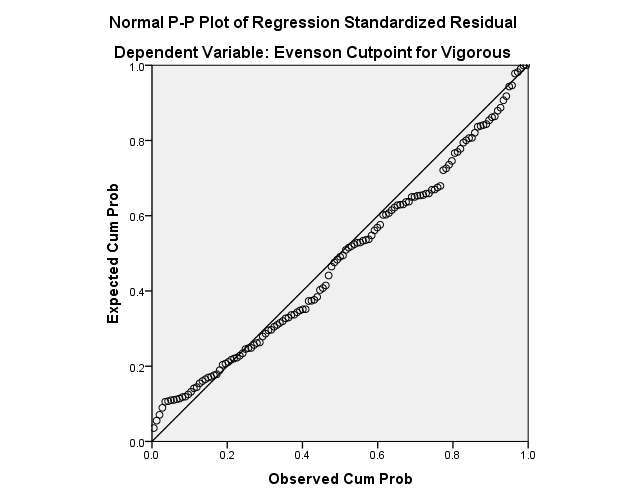


* Generalized Linear Models.

**GENLIN Num_days_Guidelines BY Internalizing gender Binary_age_10 (ORDER=DESCENDING) WITH Valid_Days**

**pwhodastotal1 pincome1 BMI_percentile**

**/MODEL Internalizing gender Binary_age_10 Valid_Days pwhodastotal1 pincome1 BMI_percentile**

INTERCEPT=YES

DISTRIBUTION=POISSON LINK=LOG

/CRITERIA METHOD=FISHER(1) SCALE=1 COVB=MODEL MAXITERATIONS=100 MAXSTEPHALVING=5

PCONVERGE=1E-006(ABSOLUTE) SINGULAR=1E-012 ANALYSISTYPE=3(WALD) CILEVEL=95 CITYPE=WALD

LIKELIHOOD=FULL

/MISSING CLASSMISSING=EXCLUDE

/PRINT CPS DESCRIPTIVES MODELINFO FIT SUMMARY SOLUTION (EXPONENTIATED).

| **Model Information** | |
| --- | --- |
| Dependent Variable | Num_days_Guidelines |
| Probability Distribution | Poisson |
| Link Function | Log |

| **Omnibus Test^a^** | | |
| --- | --- | --- |
| Likelihood Ratio Chi-Square | df | Sig. |
| 109.532 | 7 | .000 |
| Dependent Variable: Num_days_Guidelines  Model: (Intercept), Internalizing, child gender, 10 years and older, Number of Valid Days of Wear, Parent total WHODAS score, Parent's income category (before taxes), BMI_percentile^a^ | | |
| a. Compares the fitted model against the intercept-only model. | | |

REGRESSION

/MISSING LISTWISE

/STATISTICS COEFF OUTS CI(95) R ANOVA

/CRITERIA=PIN(.05) POUT(.10)

/NOORIGIN

**/DEPENDENT Evenson_Light**

**/METHOD=ENTER Externalizing Binary_age_10 Valid_Days gender pincome1 pwhodastotal1 BMI_percentile**

/SCATTERPLOT=(*SDRESID ,*ADJPRED)

/RESIDUALS HISTOGRAM(ZRESID) NORMPROB(ZRESID).

| **Model Summary** | | | | |
| --- | --- | --- | --- | --- |
| Model | R | R Square | Adjusted R Square | Std. Error of the Estimate |
| 1 | .668^a^ | .447 | .415 | 33.788475590371290 |
| a. Predictors: (Constant), BMI_percentile, Externalizing, Number of Valid Days of Wear, 10 years and older, Parent's income category (before taxes), child gender, Parent total WHODAS score | | | | |

| **ANOVA^a^** | | | | | | |
| --- | --- | --- | --- | --- | --- | --- |
| Model | | Sum of Squares | df | Mean Square | F | Sig. |
| 1 | Regression | 113396.565 | 7 | 16199.509 | 14.189 | .000^b^ |
|  | Residual | 140424.313 | 123 | 1141.661 |  |  |
|  | Total | 253820.878 | 130 |  |  |  |
| a. Dependent Variable: Evenson Cutpoint for Light | | | | | | |
| b. Predictors: (Constant), BMI_percentile, Externalizing, Number of Valid Days of Wear, 10 years and older, Parent's income category (before taxes), child gender, Parent total WHODAS score | | | | | | |

| Model | | Collinearity Statistics | |
| --- | --- | --- | --- |
|  |  | Tolerance | VIF |
| 1 | (Constant) |  |  |
|  | Parent total WHODAS score | .667 | 1.500 |
|  | BMI_percentile | .830 | 1.204 |
|  | Parent's income category (before taxes) | .792 | 1.262 |
|  | child gender | .872 | 1.147 |
|  | Number of Valid Days of Wear | .916 | 1.092 |
|  | 10 years and older | .948 | 1.055 |
|  | Externalizing | .788 | 1.269 |


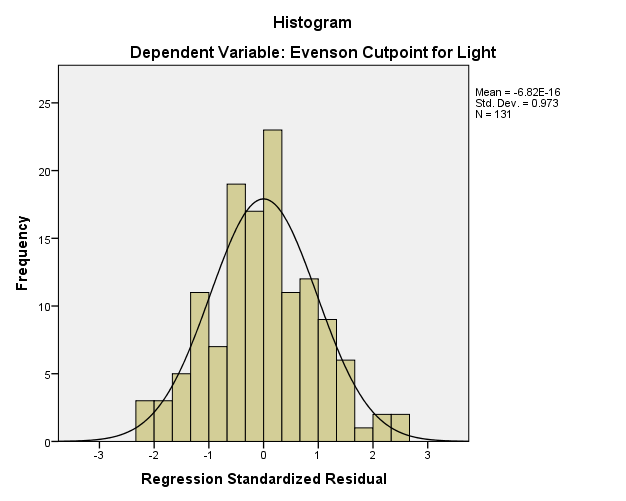


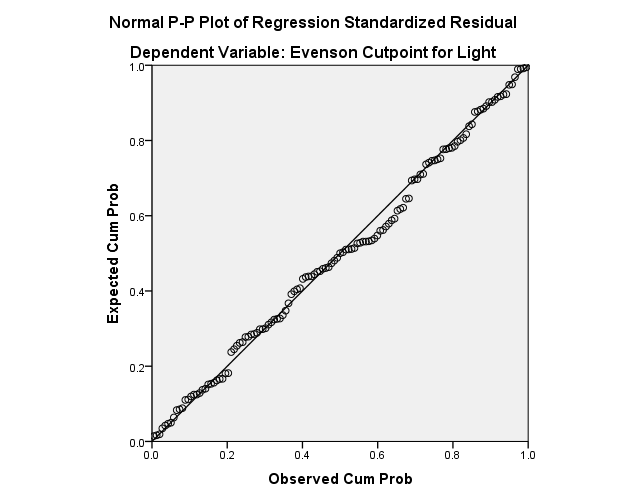


REGRESSION

/MISSING LISTWISE

/STATISTICS COEFF OUTS CI(95) R ANOVA

/CRITERIA=PIN(.05) POUT(.10)

/NOORIGIN

/**DEPENDENT Evenson_Moderate**

**/METHOD=ENTER Externalizing Binary_age_10 Valid_Days gender pincome1 pwhodastotal1 BMI_percentile**

/SCATTERPLOT=(*SDRESID ,*ADJPRED)

/RESIDUALS HISTOGRAM(ZRESID) NORMPROB(ZRESID).

| **Model Summary** | | | | |
| --- | --- | --- | --- | --- |
| Model | R | R Square | Adjusted R Square | Std. Error of the Estimate |
| 1 | .491^a^ | .242 | .198 | 8.893111618749813 |
| a. Predictors: (Constant), BMI_percentile, Externalizing, Number of Valid Days of Wear, 10 years and older, Parent's income category (before taxes), child gender, Parent total WHODAS score | | | | |

| **ANOVA^a^** | | | | | | |
| --- | --- | --- | --- | --- | --- | --- |
| Model | | Sum of Squares | df | Mean Square | F | Sig. |
| 1 | Regression | 3098.050 | 7 | 442.579 | 5.596 | .000^b^ |
|  | Residual | 9727.754 | 123 | 79.087 |  |  |
|  | Total | 12825.804 | 130 |  |  |  |
| a. Dependent Variable: Evenson Cutpoint for Moderate | | | | | | |
| b. Predictors: (Constant), BMI_percentile, Externalizing, Number of Valid Days of Wear, 10 years and older, Parent's income category (before taxes), child gender, Parent total WHODAS score | | | | | | |

| Model | | Collinearity Statistics | |
| --- | --- | --- | --- |
|  |  | Tolerance | VIF |
| 1 | (Constant) |  |  |
|  | Parent total WHODAS score | .667 | 1.500 |
|  | BMI_percentile | .830 | 1.204 |
|  | Parent's income category (before taxes) | .792 | 1.262 |
|  | child gender | .872 | 1.147 |
|  | Number of Valid Days of Wear | .916 | 1.092 |
|  | 10 years and older | .948 | 1.055 |
|  | Externalizing | .788 | 1.269 |


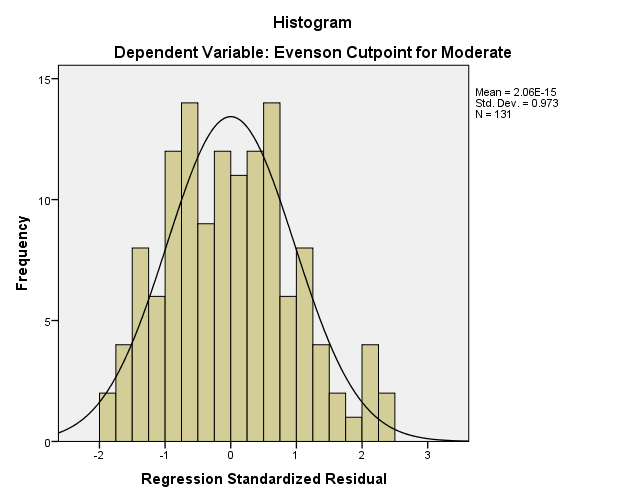


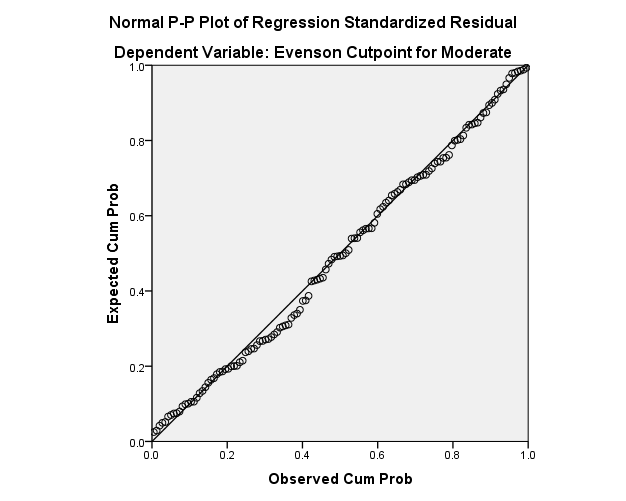


REGRESSION

/MISSING LISTWISE

/STATISTICS COEFF OUTS CI(95) R ANOVA

/CRITERIA=PIN(.05) POUT(.10)

/NOORIGIN

/**DEPENDENT Evenson_Vigorous**

**/METHOD=ENTER Externalizing Binary_age_10 Valid_Days gender pincome1 pwhodastotal1 BMI_percentile**

/SCATTERPLOT=(*SDRESID ,*ADJPRED)

/RESIDUALS HISTOGRAM(ZRESID) NORMPROB(ZRESID).

| **Model Summary** | | | | |
| --- | --- | --- | --- | --- |
| Model | R | R Square | Adjusted R Square | Std. Error of the Estimate |
| 1 | .486^a^ | .236 | .193 | 11.345012149597087 |
| a. Predictors: (Constant), BMI_percentile, Externalizing, Number of Valid Days of Wear, 10 years and older, Parent's income category (before taxes), child gender, Parent total WHODAS score | | | | |

| **ANOVA^a^** | | | | | | |
| --- | --- | --- | --- | --- | --- | --- |
| Model | | Sum of Squares | df | Mean Square | F | Sig. |
| 1 | Regression | 4895.747 | 7 | 699.392 | 5.434 | .000^b^ |
|  | Residual | 15831.244 | 123 | 128.709 |  |  |
|  | Total | 20726.991 | 130 |  |  |  |
| a. Dependent Variable: Evenson Cutpoint for Vigorous | | | | | | |
| b. Predictors: (Constant), BMI_percentile, Externalizing, Number of Valid Days of Wear, 10 years and older, Parent's income category (before taxes), child gender, Parent total WHODAS score | | | | | | |

| Model | | Collinearity Statistics | |
| --- | --- | --- | --- |
|  |  | Tolerance | VIF |
| 1 | (Constant) |  |  |
|  | Parent total WHODAS score | .667 | 1.500 |
|  | BMI_percentile | .830 | 1.204 |
|  | Parent's income category (before taxes) | .792 | 1.262 |
|  | child gender | .872 | 1.147 |
|  | Number of Valid Days of Wear | .916 | 1.092 |
|  | 10 years and older | .948 | 1.055 |
|  | Externalizing | .788 | 1.269 |


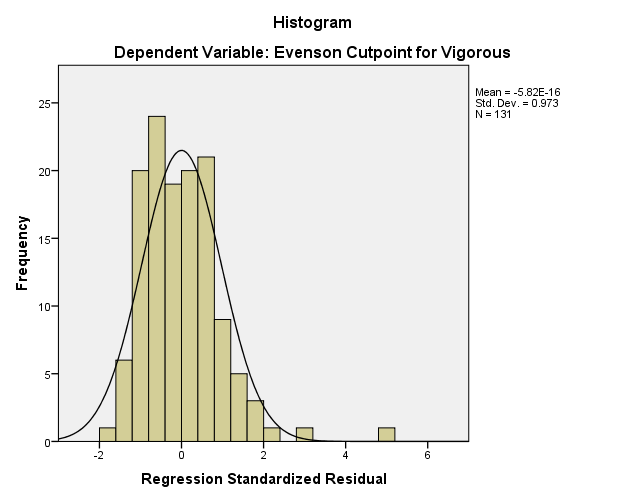


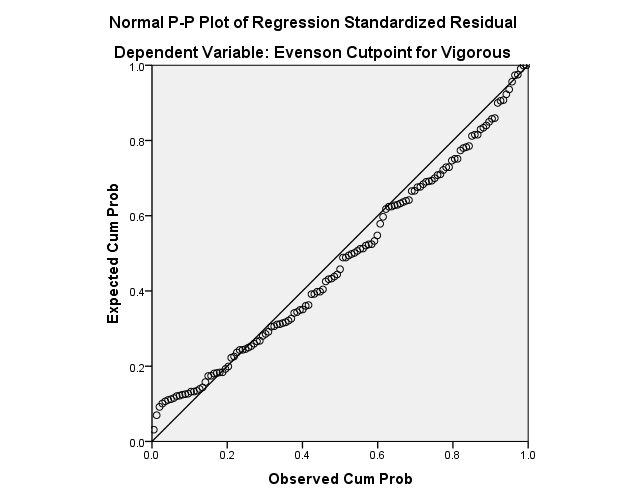


* Generalized Linear Models.

**GENLIN Num_days_Guidelines BY Externalizing gender Binary_age_10 (ORDER=DESCENDING) WITH Valid_Days**

**pwhodastotal1 pincome1 BMI_percentile**

/MODEL gender Binary_age_10 Valid_Days pwhodastotal1 pincome1 Externalizing BMI_percentile

INTERCEPT=YES

DISTRIBUTION=POISSON LINK=LOG

/CRITERIA METHOD=FISHER(1) SCALE=1 COVB=MODEL MAXITERATIONS=100 MAXSTEPHALVING=5

PCONVERGE=1E-006(ABSOLUTE) SINGULAR=1E-012 ANALYSISTYPE=3(WALD) CILEVEL=95 CITYPE=WALD

LIKELIHOOD=FULL

/MISSING CLASSMISSING=EXCLUDE

/PRINT CPS DESCRIPTIVES MODELINFO FIT SUMMARY SOLUTION (EXPONENTIATED).

| **Model Information** | |
| --- | --- |
| Dependent Variable | Num_days_Guidelines |
| Probability Distribution | Poisson |
| Link Function | Log |

| **Omnibus Test^a^** | | |
| --- | --- | --- |
| Likelihood Ratio Chi-Square | df | Sig. |
| 105.111 | 7 | .000 |
| Dependent Variable: Num_days_Guidelines  Model: (Intercept), child gender, 10 years and older, Number of Valid Days of Wear, Parent total WHODAS score, Parent's income category (before taxes), Externalizing, BMI_percentile^a^ | | |
| a. Compares the fitted model against the intercept-only model. | | |

Tests of Skewness

| **Descriptive Statistics** | | | | | |
| --- | --- | --- | --- | --- | --- |
|  | N | Mean | Std. Deviation | Skewness | |
|  | Statistic | Statistic | Statistic | Statistic | Std. Error |
| Evenson Cutpoint for Light | 140 | 165.155397109081720 | 43.255406472119260 | -.231 | .205 |
| Evenson Cutpoint for Moderate | 140 | 32.453198554398040 | 9.858792862671974 | .043 | .205 |
| Evenson Cutpoint for Vigorous | 140 | 23.761819302697380 | 12.475117525712799 | .804 | .205 |
| Valid N (listwise) | 140 |  |  |  |  |
